# Individual Differences and State-Dependent Responses in Transcranial Direct Current Stimulation

**DOI:** 10.3389/fnhum.2016.00643

**Published:** 2016-12-21

**Authors:** Tzu-Yu Hsu, Chi-Hung Juan, Philip Tseng

**Affiliations:** ^1^Research Center of Brain and Consciousness, College of Humanities and Social Sciences, Taipei Medical UniversityTaipei, Taiwan; ^2^Shuang-Ho Hospital, Taipei Medical UniversityNew Taipei City, Taiwan; ^3^Graduate Institute of Health and Biotechnology Law, Taipei Medical UniversityTaipei, Taiwan; ^4^Institute of Cognitive Neuroscience, National Central UniversityTaoyuan, Taiwan; ^5^Graduate Institute of Humanities in Medicine, Taipei Medical UniversityTaipei, Taiwan

**Keywords:** non-invasive brain stimulation, transcranial direct current stimulation (tDCS), visual working memory (VWM), state-dependence, right posterior parietal cortex (rPPC)

## Abstract

Transcranial direct current stimulation (tDCS) has been extensively used to examine whether neural activities can be selectively increased or decreased with manipulations of current polarity. Recently, the field has reevaluated the traditional anodal-increase and cathodal-decrease assumption due to the growing number of mixed findings that report the effects of the opposite directions. Therefore, the directionality of tDCS polarities and how it affects each individual still remain unclear. In this study, we used a visual working memory (VWM) paradigm and systematically manipulated tDCS polarities, types of different independent baseline measures, and task difficulty to investigate how these factors interact to determine the outcome effect of tDCS. We observed that only low-performers, as defined by their no-tDCS corsi block tapping (CBT) performance, persistently showed a decrement in VWM performance after anodal stimulation, whereas no tDCS effect was found when participants were divided by their performance in digit span. In addition, only the optimal level of task difficulty revealed any significant tDCS effect. All these findings were consistent across different blocks, suggesting that the tDCS effect was stable across a short period of time. Lastly, there was a high degree of intra-individual consistency in one’s responsiveness to tDCS, namely that participants who showed positive or negative effect to anodal stimulation are also more likely to show the same direction of effects for cathodal stimulation. Together, these findings imply that tDCS effect is interactive and state dependent: task difficulty and consistent individual differences modulate one’s responsiveness to tDCS, while researchers’ choices of independent behavioral baseline measures can also critically affect how the effect of tDCS is evaluated. These factors together are likely the key contributors to the wide range of “noises” in tDCS effects between individuals, between stimulation protocols, and between different studies in the literature. Future studies using tDCS, and possibly tACS, should take such state-dependent condition in tDCS responsiveness into account.

## Introduction

Transcranial direct current stimulation (tDCS) is a non-invasive stimulation technique, and its therapeutic and neuronal-based enhancing potential has attracted interest from basic scientists and clinicians alike. By applying a weak electric current over the scalp, where cortical neuronal activities beneath the stimulated area would change with the direction of current flow, tDCS can modulate cortical excitability and, consequently, various cognitive performances. Early animal studies have reported a bi-directional effect of tDCS in modulating neural activities, where anodal tDCS was associated with the depolarization of neurons, and cathodal tDCS was associated with the hyperpolarization of neurons (Creutzfeldt et al., 1962; Bindman et al., 1964; Purpura and McMurtry, 1965). Similar effects were also observed in humans’ motor cortex excitability (Nitsche and Paulus, 2000; Stagg and Nitsche, 2011; Pellicciari et al., 2013), where anodal and cathodal stimulation increased and decreased MEP amplitudes, respectively. This suggests that, consistent with previous studies done on animals, the excitability of cortico-motor neurons was modulated by the current direction of tDCS (Antal et al., 2007; Miyaguchi et al., 2013; Chew et al., 2015). The assumption of bipolarity with opposite neuronal and cognitive effects has since been adopted in many of the earlier cognitive work (for a review, see Paulus, 2011; Vallar and Bolognini, 2011; Jacobson et al., 2012; Horvath et al., 2015a,b). For example, anodal stimulation over the left dorsolateral prefrontal cortex (DLPFC) increased the number of correct responses in a 3-back working memory (WM) task (Fregni et al., 2005). Along the same line, anodal stimulation over the left DLPFC also improved WM performance (Zaehle et al., 2011) and decreased reaction times (Mulquiney et al., 2011) whereas no improvement/decrement on memory performance was observed after cathodal stimulation on the same brain area (Fregni et al., 2005; Ohn et al., 2008; Andrews et al., 2011; Zaehle et al., 2011). Additionally, tDCS has also revealed its great potential in treatment. Improvement in major depression (Fregni et al., 2006; Brunoni et al., 2011), memory deficit in Parkinson disease (Boggio et al., 2006a,b), aphasia (Baker et al., 2010; Kang and Paik, 2011; You et al., 2011) and recovery from stroke patients (Fregni et al., 2005; Miniussi et al., 2008; Jo et al., 2009; Kang et al., 2009; Bolognini et al., 2011; Bueno et al., 2011) all suggest that neuromodulation is able to critically affect patients’ cognitive functions.

Recently, despite the simple anodal-increase and cathodal-decrease rules of thumb, many studies have observed that, beyond tDCS polarity, stimulation parameters such as duration, intensity, frequency, electrode position and control settings can also modulate the final outcome of the tDCS effect (Teo et al., 2011; Jacobson et al., 2012; Batsikadze et al., 2013; Brunoni et al., 2013; Hoy et al., 2013; Benwell et al., 2015; Horvath et al., 2015a,b). In addition, inter- and intra-individual differences, including genetics, age, gender, physiological differences and baseline task performances, all imply the importance of “neural state” that may determine the modulating effect through its interaction with tDCS (Mattay et al., 2003; Cheeran et al., 2008; Krause and Cohen Kadosh, 2014; Veniero et al., 2016). Supporting evidence from pharmacologic studies showed that L-dopa-induced learning and memory formation can interact with tDCS-induced neuroplasticity (Monte-Silva et al., 2010). When L-dopa was applied alone, the dosage of dopamine and cognitive functions displayed an inverted U-shaped relationship: mainly, when dosage of L-dopa was low or high, the corresponding plasticity was inhibited, whereas a medium dosage facilitated neural plasticity. When tDCS was applied concurrently with medium dosage of L-dopa, tDCS turned facilitatory plasticity into inhibitory, suggesting that tDCS induced plasticity changes in a similar fashion as L-dopa. Also, this possibly suggests that tDCS might have placed an additive/subtractive effect to the medium dosage of L-dopa, which turned median dosage into low/high dosage to induce such inhibitory effect.

The non-linear state-dependence of the tDCS effect was not only found in pharmacological studies, but also in cognitive performances (Learmonth et al., 2015). In the field of visual working memory (VWM), previous fMRI studies have reported that the BOLD signal of the posterior parietal cortex (PPC) would increase with memory capacity until reaching a neural and behavioral plateau (Todd and Marois, 2004; Vogel et al., 2005; Xu and Chun, 2006). According to the anodal-increase and cathodal-decrease rules of thumb, anodal stimulation over PPC should increase neural activities and memory performance, and vice versa for the cathodal stimulation. However, the observed effect of tDCS was much more complicated. Our previous studies reported that, despite identical stimulation parameters and proper counterbalancing, the effect of tDCS was not equal for all participants: it altered with participants’ baseline performance. When we lined up the participants based on their natural performances from the sham-tDCS condition, only low performers showed a boost in neural activities and behavioral WM performance with right PPC (rPPC) anodal stimulation, but not the high performers (Tseng et al., 2012). Evidence from behavioral, event-related potentials, and alpha oscillation all supported the finding that memory capacity in low performers was selectively enhanced by rPPC anodal stimulation (Tseng et al., 2012; Hsu et al., 2014). The same pattern was also observed when we used AC stimulation in combination with a similar VWM task (Tseng et al., 2016). These findings suggest that the baseline state of each individual is different and that the tDCS effect, or one’s receptivity to the tDCS effect, changes with his or her baseline performance. Together, this observation is also consistent with the transcranial magnetic stimulation (TMS) literature, suggesting that the effect of tDCS is associated with the neural state of the stimulated individuals (Dockery et al., 2009; Berryhill and Jones, 2012; Tseng et al., 2012; Hsu et al., 2014; Benwell et al., 2015; Learmonth et al., 2015).

Given the growing number of studies reporting varied effects of tDCS with different baseline performances (Gözenman and Berryhill, 2016; Heinen et al., 2016; Looi et al., 2016), it is important for studies to choose an appropriate baseline on which to evaluate the effect of tDCS. The studies mentioned above have mostly adopted participants’ behavioral performances from the sham condition to serve as a baseline to split the participants into different groups (Tseng et al., 2012, 2016; Hsu et al., 2014). Alternatively, one important study by Jones and Berryhill (2012) adopted the digit span task as an independent measure, rather than using VWM performance from the sham condition, to split the participants into low and high performers. The authors found that anodal stimulation increased memory performance. More importantly, they also found that the tDCS effect varied with participants’ baseline digit span performance: only the high WM capacity group enjoyed an improvement after stimulation but not the low WM capacity group. Recently, Heinen et al. (2016) also provided another evidence of tDCS effect varying with participants’ baseline performance. They showed that only cathodal stimulation enhanced WM precision, especially for those participants whose baseline performance was low. Together, although these studies are not entirely consistent with one another, they do point out one thing in common: the importance of baseline memory performance on which to evaluate tDCS effect.

In addition to baseline performance, task difficulty is also another likely contributor to the state-dependent nature of the effects of tDCS. In a cognitive control task, the effect of tDCS was observed in the easy and medium difficulty conditions, but not the most difficult condition. In the context of VWM, Jones and Berryhill (2012) found that only the high performers showed improved memory performance with tDCS as task difficulty increased. Using another VWM paradigm, Wu et al. (2014) also found that the most difficult memory condition is usually the one that participants show a significant amount of tDCS-induced improvement. However, without using the same memory paradigm, it is difficult to equate or properly compare task difficulties across studies.

Based on the mixed findings reviewed above, the present study aims to investigate the interaction between tDCS polarity, task difficulty, and individual differences by systematically varying different parameters of task difficulty and tDCS polarity, while testing them on the same set of individuals that include a mixture of low and high performers. We continued to use a VWM change detection task since VWM has been extensively investigated with tDCS, thus better relevance with the existing literature. For a better understanding of the influence of baseline performance on tDCS effects, we also revisited the issue of splitting participants by using the digit span task and another visuospatial WM variant—the corsi block tapping (CBT) task. CBT is a well-studied task, and is widely used in the clinical population to evaluate their VWM performance (e.g., Kessels et al., 2000). Furthermore, it has been shown to be sensitive to anodal tDCS both in the healthy (Wu et al., 2014) and neurological (Wu et al., 2016) populations. In the present study, participants performed digit span and CBT, in counterbalanced order, on a separate day prior to their participation in the formal session, which included sham, anodal and cathodal tDCS on three different days (separated by a week) in counterbalanced order. Finally, we analyzed participants’ change detection performance, the main dependent measure of this experiment, in two different ways. We approached this by splitting the participants either based on their CBT or digit span performance. Levels of task difficulty were also included to investigate whether the effects of tDCS would change with task difficulty.

## Materials and Methods

### Ethical Standards

This study has received the human study approval (101-1930A3) from the Institutional Review Board, Linkou Chang Gung Memorial Hospital, Taoyuan County. It has been carried out in accordance with The Code of Ethics of the World Medical Association (Declaration of Helsinki).

### Participants

Eighteen right-handed participants (mean age 22.7 years, range of 20–27; 11 females and 7 males) were recruited in this experiment. All had normal or corrected-to-normal visual acuity, and reported no neurological history. All participants signed informed consent prior to their participation in the experiment and they received monetary reimbursement upon completion of all four sessions (one behavioral pre-session and three tDCS sessions).

### Apparatus

Stimuli were presented on a 19-inch CRT screen using a video resolution of 1024 × 768 pixels and a vertical refresh rate of 100 Hz. Subjects sat 57 cm in front of the screen, which was positioned at eye level. Stimuli were generated and delivered in MATLAB (MathWorks) using Psychtoolbox (Brainard, 1997; Pelli, 1997), which controlled the presentation of the stimuli and recorded participants’ responses.

tDCS was delivered with a Magstim Eldith DC-stimulator and a pair of electrodes housed in 4 cm × 4 cm saline-soaked sponge coverings. The center of the stimulation electrode was placed over the target site, P4 according to the international 10-20 system for EEG electrode placement. P4 was chosen because of its importance in the task used in the present study (Vogel et al., 2005; for a review, see Juan et al., in press), and also because of our goal of comparing against previous tDCS studies that have investigated the effects of tDCS in change detection performances (Jones and Berryhill, 2012; Tseng et al., 2012; Hsu et al., 2014). The other electrode was placed over the left cheek. In the tDCS conditions the current was applied for 15 min with an intensity of 1.5 mA (Berryhill et al., 2010; Jones and Berryhill, 2012; Tseng et al., 2012; Hsu et al., 2014). The sham tDCS condition followed an identical procedure, including electrode placements, but only ramp-up and ramp-down for a total of 30 s and no electric stimulation for 15 min.

### Design and Procedure

The entire experiment consisted of four separate sessions: the behavioral pre-session, sham tDCS, anodal tDCS and cathodal tDCS. The behavioral pre-session always took place on the first day, while the order of the three tDCS sessions were counterbalanced across participants. Each tDCS session was separated for at least 1 week apart to control for any unanticipated carry-over effects. On the day of the behavioral pre-session, participants completed a block of digit span and CBT in counterbalanced order. On the remaining 3 days, participants first completed 36 practice trials of the change detection task, then went through 15 min of tDCS, and finally completed 576 more trials of change detection task over the course of eight blocks.

Each participant was to complete a computer-based version of CBT task (Corsi, 1972; Bo et al., 2011; Brunetti et al., 2014) to measure their baseline performance in visuospatial WM. The task requirement was that participants had to reproduce a given flash sequence by mouse-clicking on the corresponding blocks. There were nine blue colored patches on each array. Only one of them flashed with yellow color on each array for 200 ms. The experiment started with sequences of two flashes, which constituted as the easiest trial. The length of the sequence was gradually increased by one item when the participants correctly recalled the sequences on two consecutive trials. In contrast, the task ended if the participant could not reproduce the given sequences in two consecutive trials. The length of the sequence in the very last trial would serve as an index of that person’s visuospatial WM span. Participants also performed a computer-based version of forward digit span on the same day. Each digit was presented by voice with a 1 s interval between each digit. After each sequence, participants were to repeat the sequence by pressing the corresponding number keys on a keyboard. The cutoff procedure for digit span is identical to the CBT, where two consecutive correct trials would lead to a one-digit span increase, and two consecutive error trials would end the task and determine participant’s verbal WM capacity.

The experimental design included within-subjects factors of a set size (4, 6, 8), tDCS (anodal, cathodal, sham), and a between-subjects factor of groups (low performer, high performer). In the change detection task, each trial began with 1000–1500 ms fixation, followed by a 500 ms cue array, a 500 ms memory array, a 1000 ms retention interval, and a 2000 ms test array. Participants were to click the left button on the mouse with their right index finger when there was a change or click right button on the mouse with their right middle finger for no change. The task was modified from the Vogel and Machizawa (2004) study. All the stimuli were presented within two 5° × 12° rectangular regions placed 1° away from a central fixation cross on a gray background. Each memory array consisted of 4, 6, or 8 colored squares (0.4° × 0.4°) in each hemifield. The color of each square on memory array was randomly selected from a set of colors (red, green, blue, yellow, dark gray, pink, purple, cyan and white). Stimulus positioning was randomized on each trial. In 50% of trials, one of the colored squares in the test array would differ from the memory array (also known as change trials), with the remaining 50% of trials being no-change trials. Before the memory array, a central arrow cue would instruct the participants to remember the items in either the left or right hemifield.

### Data Analyses

Two measures were used to index participants’ performance: *d*′ and Pashler’s K. The value of *d*′ is a common measure of sensitivity derived from the signal detection theory (Macmillan and Creelman, 1991). The *d*′ is estimated by the difference of standardized hit rate and false alarm rate (1). Larger *d*′ means higher sensitivity whereas *d*′ near zero means chance-level performance. Pashler (1988) *K* is a formula used in estimating how many items are held in one’s memory (2). The rationale is that if an individual can hold *K* number of items in memory from an array out of S items, then *K* could be estimated via set size and correct response rate to change trials. To correct for guessing and interference from the test array, false alarm rate is also taken into account in the formula (Rouder et al., 2011).

(1) $$d^{\prime }\;=\;z\left(\text{Hit}\;\text{rate}\right)-z\left(\text{False}\;\text{alarm}\;\text{rate}\right);$$

(2) $$K\;=\;\frac{\text{Set}\;\text{Size}\;\text{*}\;(\text{Hit}\;\text{rate}\;\text{-}\;\text{False}\;\text{alarm}\;\text{rate})}{\left(\text{1}\;\text{-}\;\text{False}\;\text{alarm}\;\text{rate}\right)}$$

## Results

### Individual Differences in Responsiveness to tDCS

As other studies have previously documented (Chew et al., 2015), there was a wide range of individual differences even at set size 4 where the difficulty level is optimal (see our analysis in the sections below). However, although differences existed between different individuals (Figure 2, left chart), the directions of tDCS effect seemed quite consistent within each individual. That is, when we computed the anodal-sham and cathodal-sham contrasts for each participant, most of the participants had the same direction of tDCS effect for both anodal and cathodal stimulation. In summary, there were seven participants who showed improvement in both anodal and cathodal sessions, five participants who showed consistent impairment regardless of tDCS polarity, and five participants whose tDCS performance followed the traditional anodal-increase and cathodal-decrease assumption (Figure 2, lower right pie chart). Only one participant showed a cathodal-increase and anodal-decrease pattern that is less consistent with the literature.

At the group level, from the assumption of anodal-increase and cathodal-decrease, a negative correlation between anodal-increased performance and cathodal-decreased performance would be expected. To investigate whether this assumption also applies to the current study, a correlation analysis was conducted on signal detection performance between anodal-sham and cathodal-sham contrasts. However, a positive correlation between anodal-sham and cathodal-sham was found, *r*_(18)_ = 0.692, *p* = 0.001, which is not supporting the anodal-increase and cathodal-decrease assumption. In addition, Pearson’s chi-square test was conducted to examine whether the tDCS effect on each individual was coming from the same distribution or not. The tDCS effect was relabeled according to the size of contrast scores. When the contrast scores are smaller or equal to ±0.5, they are relabeled as ±1. When the contrast scores larger than ±0.5, which is quite large in terms of *d*′, the contrast scores are relabeled as ±2. The result showed that the tDCS effect on each individual was coming from different distributions, ${\text{χ}}_{\left(9,\;N\;=\;18\right)}^{\text{2}}$ = 24.05, *p* = 0.004. This can potentially be explained by the high intra-subject consistency between anodal and cathodal tDCS described above.

### Splitting Participants into Low- and High-Performing Groups Using Forward Digit Span Task

Participants were split into two groups according to their digit span score. Independent sample *t*-test showed a significant group differences, *t*_(16)_ = −4.24, *p* = 0.001. The digit span score was significantly higher in the high-performing group (*M* = 10.11) than the low-performing group (*M* = 7.88). To check whether our statistical power was undermined by limited sample size, we conducted *post hoc* power analyses using GPower (Faul and Erdfelder, 1992; for a full description, see Erdfelder et al., 1996) with effect size *d* = 2.006, power (1 − β) set at 0.95 and *α* = 0.05, two-tailed. The analysis showed that power reaches 0.963 when sample sizes are 8 and 8 for group 1 and 2 for group differences to reach statistical significance at the 0.05 level. Thus, our sample sizes even after dividing participants into subgroups do not seem to compromise statistical power too much. This “group” factor also was integrated into subsequence analysis. Three-way mixed effect ANOVA was conducted to investigate the effects of groups (low vs. high), tDCS (anodal, cathodal, sham) and set size (4, 6, 8) on the behavioral indexes: *d*′ and *K*. The *d*′ data showed significant main effects of set size, *F*_(2,32)_ = 118.63, *p* = 0.000, $\eta_{p}^{2}$ = 0.881. No other main effects or interactions reached significance (*p* > 0.05).

Regarding *K* values, the main effect of tDCS, *F*_(2,32)_ = 0.737, *p* = 0.487, $\eta_{p}^{2}$ = 0.044, and set size, *F*_(2,32)_ = 0.639, *p* = 0.534, $\eta_{p}^{2}$ = 0.038, both did not reach statistical significance. Only the interaction between set size and group did, *F*_(2,32)_ = 3.879, *p* = 0.031, $\eta_{p}^{2}$ = 0.195, because *K* values in set size 6 was significantly higher than those in size set 8 (*p* = 0.008). No other comparisons showed any significant difference. These results suggest that perhaps digit span is not an optimal measure to divide participants’ visuospatial WM performance. Participants who had high digit span scores did not have high change detection performance, and vice versa for low performers, suggesting that digit span is probably not tapping into the same mechanisms used by VWM.

### Splitting Participants into Low- and High-Performing Groups Using CBT Task

We also divided participants into low and high performers based on their CBT performance. Independent sample *t*-test showed significant group differences, *t*_(16)_ = −5.030, *p* = 0.000, where the high performers (*M* = 7.55) significantly outperformed low performers (*M* = 5.88). We again conducted *post hoc* power analyses using GPower (Faul and Erdfelder, 1992; for a full description, see Erdfelder et al., 1996) with effect size *d* = 2.507, power (1 − β) set at 0.95 and *α* = 0.05, two-tailed. The analysis showed that power reaches 0.973 when sample sizes are 6 and 6 for group 1 and 2 for group differences to reach statistical significance at the 0.05 level. This “group” factor was also integrated into subsequence analysis. Three-way mixed effect ANOVA was conducted to investigate the effects of groups (low vs. high), tDCS (anodal, cathodal, sham) and set size (4, 6, 8) on the behavioral indexes: *d*′ and *K*. The *d*′ data showed significant main effects of set size, *F*_(2,32)_ = 158.366, *p* = 0.000, $\eta_{p}^{2}$ = 0.908, along with a significant interaction between set size and group, *F*_(2,32)_ = 5.379, *p* = 0.010, $\eta_{p}^{2}$ = 0.252. The *d*′ scores in set size 4 condition were significantly higher than those under set size 6, which in turn was higher than set size 8 (*p*s < 0.01). Participants’ performance significantly decreased with increasing set sizes. The interaction arose because low performers’ *d*′ was significantly lower than high performers under set size 4 (*p* = 0.040), with no group difference for set size 6 or 8 (*p*s > 0.05), suggesting low and high performers showed different target detection abilities only under relatively-easy condition. No other main effects or interactions reached statistical significance (*p* > 0.05).

Regarding *K* values, the main effect of tDCS, *F*_(2,32)_ = 0.852, *p* = 0.436, $\eta_{p}^{2}$ = 0.051, and set size, *F*_(2,32)_ = 0.514, *p* = 0.603, $\eta_{p}^{2}$ = 0.031, were not significant. A marginally-significant interaction between tDCS and group was observed, *F*_(2,32)_ = 3.066, *p* = 0.061, $\eta_{p}^{2}$ = 0.161. The simple main effect showed that K values for low performers in the anodal condition was significantly lower than those in the sham condition (*p* = 0.01), and marginally lower than those in the cathodal condition (*p* = 0.069). A significant interaction between tDCS, set size, and group was observed, *F*_(4,64)_ = 2.502, *p* = 0.050, $\eta_{p}^{2}$ = 0.135. The interaction arose because *K* value in the anodal condition was lower than those in the sham condition under set size 6 (*p* = 0.032) and 8 (*p* = 0.045) within low performers, but not high performers, indicating that anodal stimulation selectively interfered low performers’ memory performance in the more difficult conditions (Figure 3). Other comparisons were not statistically significant.

To further investigate the stability of tDCS effect across time in low performers, another three-way repeated measure ANOVA was conducted to examine the effect of tDCS, set size, blocks. A significant main effect of tDCS was observed, *F*_(2,16)_ = 4.354, *p* = 0.031, $\eta_{p}^{2}$ = 0.352. *Post hoc* analysis showed that *K* values in the anodal condition was lower than those in sham (*p* = 0.029) and cathodal (*p* = 0.073) conditions across time, indicating that anodal stimulation constantly affected VWM performance across different blocks, rather than being modulated by extreme cases.

**Figure 1:**
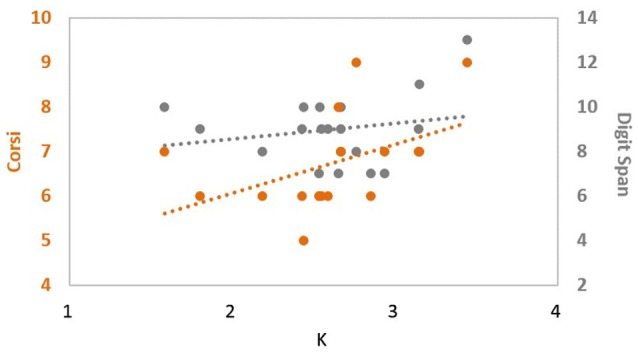
**Correlation between change detection performance and different independent measures.** corsi block tapping (CBT) scores (orange) showed a significant positive correlation with participants’ change detection performance (*X*-axis), while digit span (gray) did not, suggesting that CBT and digit span tasks are most likely probing different mechanisms of memory.

Given these results in set size 4, we conducted another correlation analysis to see whether there was any correlation between the different choices of independent baseline measure and the dependent measure. There was a significant correlation between CBT and *K*, *ρ*_(18)_ = 0.606, *p* = 0.008. In contrast, no significant correlation was observed between digit span scores and *K* under the same condition, *ρ*_(18)_ = 0.051, *p* = 0.841. These suggest that the processing of digit span and change detection likely relies on different mechanisms (Figure 1) and highlights the fact that an independent VWM measure can dissociate high and low performing groups on a near-transfer VWM task better than an independent far-transfer verbal WM measure.

## General Discussion

The aim of the present study was to investigate the interaction between different choices of independent baseline measures, task difficulty, individual differences and tDCS polarity. Here is a brief summary of our findings regarding each factor. In terms of independent baseline measures, when we divided participants using CBT, we observed an impairment effect from anodal tDCS only in the low performers, while high performers’ WM capacity remained unaltered. No significant results were observed if we used digit span to separate participants. Therefore, choices of independent behavioral measures are indeed critical to the interpretation and analysis of the effects of tDCS. In terms of task difficulty, we found that set size 4, where participants are properly challenged but have not hit floor performance, is the optimal level of difficulty for the effect of tDCS to show through. Regarding individual differences and tDCS polarity, there was a high degree of intra-subject consistency in the direction of tDCS effects, and one-thirds of participants who showed anodal-increase/cathodal-decrease trends that are consistent with the literature, suggesting that the traditional assumption may perhaps be valid, but only applies to a subset of participants, where most of the participants respond the same way to both anodal and cathodal stimulation. We discuss each of these points in more details below.

### Implications for Choosing Independent Behavioral Baseline Tasks for tDCS Studies

We obtained similar results as previous findings on alternating VWM performance through tDCS (Jones and Berryhill, 2012; Tseng et al., 2012; Berryhill et al., 2014; Hsu et al., 2014). Participants were split by their performance in sham condition (Tseng et al., 2012; Hsu et al., 2014) or by independent CBT task, and both approaches showed a tDCS effect in elevating low performers’ memory performance. There was no correlation between digit span and change detection performance, thus digit span may be probing different neural and cognitive mechanisms from VWM. Together, these results suggest that, regardless of using the sham baseline or another independent measure such as the CBT, as long as the baseline is something similar to the dependent task measures (evidenced by significant positive correlation), the effect of tDCS can be quite evident and it is usually the low performers that are more responsive to such effect. This pattern cannot be explained by regression to the mean because no VWM studies to date have reported a declining effect in the high performers. Thus the responsiveness to tDCS in low performers seems quite specific. Similarly, the effective polarity also seems quite specific: previous students and the current experiment have all shown effective stimulation via anodal tDCS, and no effect was found with cathodal stimulation in both low and high performers. This helps rule out the factor of poor motivation (Berryhill et al., 2014), which would predict an equal, or randomly distributed, improvement effect that is not specific to anodal tDCS only.

### tDCS, rPPC and Visual WM

One notable difference between the present and previous studies is the direction of the effect of anodal tDCS on VWM. Previous studies (Tseng et al., 2012; Hsu et al., 2014) have consistently reported that anodal stimulation improved low performers’ VWM performance, and Tseng et al. (2012) proposed that this may be due to the fact that low performers had room for increased activities (neural) and improvement (cognitive), whereas the high performers do not. In the current study, however, we found an impairing effect of anodal stimulation on the low performers, even though our low performers also had plenty of room for improvement. There are several possible explanations for this. The most notable change in the current paradigm is the addition of a directional cue that instructs participants to remember one side of stimuli while inhibiting the opposite side. This manipulation increased the role of visual attention, orienting and distractor inhibition, which is has been associated with frontal areas such as the frontal eye fields or DLPFC (e.g., Wu et al., 2014). As such, stimulating and improving one’s memory abilities non-selectively (for a similar finding in non-selective memory, see Tseng and Bridgeman, 2011) may be helpful in a conventional change detection paradigm where every stimulus is a potential target with no obvious distractors to be inhibited (Tseng et al., 2012; Hsu et al., 2014), this non-selective memory mechanism is detrimental in the current paradigm because it doubles one’s memory load when it is clearly optimal not to.

The complexity and divergent functions of any brain region obviously increases the difficulty in defining anatomical specificity for tDCS (Peterchev et al., 2012; Bikson and Rahman, 2013). Studies applying tDCS over rPPC have shown that, even with identical montage and setup, the positive effects of tDCS on cognitive functions such as WM and spatial attention (Tseng et al., 2012; Hsu et al., 2014; Wu et al., 2014, 2016; Juan et al., in press) can vary quite a bit, depending on the participants’ current task set and cognitive context. Under the sliding-scale concept (Bikson and Rahman, 2013), anodal stimulation may enhance either the subgroup of neurons for WM or spatial attention, though excitation of multiple subgroups may lead to mutual inhibition, thus impairing WM and spatial attention. This would also be consistent with the state-dependency/signal-to-noise ratio account (Silvanto et al., 2007, 2008; Miniussi et al., 2010, 2013; Ruzzoli et al., 2010; Benwell et al., 2015), which proposes that the relative balance between task relevant (“signal”) and irrelevant (“noise”) neurons at baseline has a strong impact on tDCS outcomes. However, when both subgroups of neurons are boosted simultaneously by anodal stimulation, these two subgroups of neurons may compete with each other through mutual inhibition and lead to poor performance. Lastly, another possibility is that anodal stimulation may have kept active neurons from declining, thus leading to poor performance. This trend is evident from the L-dopa study (Monte-Silva et al., 2010), where optimal cognitive functions can only be observed at medium dosage of L-dopa. Increasing the amount of L-dopa actually resulted in a decline in cognitive functioning, suggesting that extremely high or low neuronal activity is associated with poor performance. In this light, anodal stimulation may elevate PPC’s activities beyond the optimal point. However, these two speculations are beyond the scope of the current study.

Recently, one study by Heinen et al. (2016) found that cathodal stimulation over rPPC can selectively enhanced memory performance by reducing the number of misbinding errors. In addition, this was found in low-performers but not high-performers. The authors provided comprehensive details and suggested that cathodal stimulation over the PPC may enhance VWM performance by boosting the attentional selection mechanism via preventing feature-misbinding and protecting the memory trace. In contrast to our studies, these authors have consistently found improved memory performance using cathodal stimulation over rPPC (Heimrath et al., 2012; Heinen et al., 2016), with an interesting difference that our studies applied tDCS before the task while Heinen et al. (2016) applied tDCS during the task. This suggests that even the timing of tDCS application can have profound impact on the traditional assumption of tDCS polarity and its effects on cognitive functioning. When tDCS is applied before the task, all task-relevant or irrelevant neural activities are non-selectively increased until the first stimulus is finally introduced, which gave participants the proper cognitive task set that would define which stimulus to be relevant and useful for the next hour or so. This timing is obviously different in the concurrent stimulation paradigm, where the balance between task-relevant and irrelevant activities is well established at the start, which would create a different neuronal state that would interact differently with tDCS. However, the poor focality of the conventional tDCS pads is likely to result in diffused electrical current across adjacent areas of the target region (Datta et al., 2009). From one study by Datta et al. (2009), the highest electric field/current density was estimated and found in the frontal regions rather than the area beneath the stimulated site. Our montage is similar to that of Datta et al. (2009) with the exception that one patch was placed over the right instead of left parietal region. With this rationale, one potential factor is that tDCS may excite unintended frontal regions that then lead to the different findings across studies. Additionally, the asymmetrical nature of tDCS effect has been documented by many studies (e.g., Ellison et al., 2014). Here we also did not observe any cathodal-induced performance changes. The underlying mechanism behind such asymmetry still requires further investigation.

### tDCS and Task Difficulty

We have previously reported an anodal tDCS effect that improves VWM performance in low performers. In contrast, Jones and Berryhill (2012) observed an improved effect in the high performers after anodal or cathodal stimulations. In addition, in the present study we observed an impairment effect in low performers after anodal stimulation. However, note that in Jones and Berryhill’s study, participants’ mean digit span scores for each group were 10.8 for low and 14.10 for high performers, which is quite different from 7.88 and 10.11 in the current study. These numbers highlight the importance of individual differences in baseline performance as they may determine the final tDCS outcome.

Regarding task difficulty, one consistent finding across several studies (Jones and Berryhill, 2012; Wu et al., 2014, 2016) is that tDCS effect usually emerges in difficult task settings that is challenging for the participants. Indeed, across these studies, no tDCS effect was observed under set size 4 in both low or high performers. Therefore, future studies can perhaps focus on the optimal level of task difficulty, knowing that it is the most likely level at which the effect of tDCS will emerge. In terms of neural activations, it is likely that when tasks are easy, the overall activation of task-relevant and irrelevant neurons is limited such that a small tip of the balance via tDCS is hard measure. As task difficulty increases, any tiny changes to the signal-to-noise ratio would then lead to observable behavioral outcomes.

Lastly, in the present study we observed that the effect of tDCS was stable across different blocks. This suggests that the aftereffect of offline anodal tDCS over PPC can be quite persistent for at least 60–90 min or so. Furthermore, the fact that tDCS was stably observed across different blocks suggests that the tDCS effect was not caused by a specific block. Thus, the effect of tDCS may consistently affect memory performance within a given period of time.

### Individual Differences in Response to tDCS Polarity

**Figure 2:**
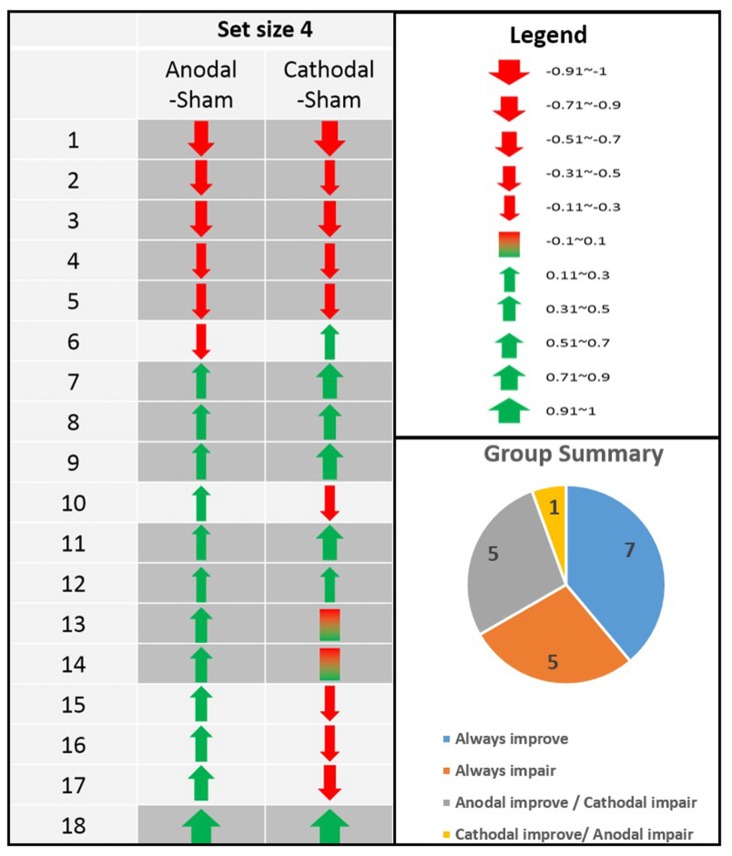
**Individual differences in the directions of tDCS effects in 18 participants (Anodal-sham and Cathodal-sham contrasts).** Even in set size 4 where the level of difficulty is optimal, there is still a wide range of individual differences. Interestingly, although differences exists between different individuals (left chart), the directions of tDCS effect is quite consistent within each individual (lower right pie chart).

One observation from the present study that is worth noting is the range of individual differences in the aftereffects of tDCS. Out of the 18 participants, there were seven participants who showed improvement in both anodal and cathodal sessions, five participants who showed consistent impairment regardless of tDCS polarity, and five participants whose tDCS performance followed the traditional anodal-increase and cathodal-decrease assumption (Figure 2, lower right pie chart), with one participant showing a cathodal-increase and anodal-decrease pattern that is less consistent with the literature. Therefore, although there is considerable *inter*-subject differences in the directions of tDCS effect, within each participant there also seems to be a high degree of *intra*-subject consistency. Twelve out of 18 participants either always showed improved performance or impaired performance following stimulation regardless of tDCS polarity. Therefore, two-thirds of our participants seemed to be insensitive to polarity manipulation. Of the remaining one-third, five out of six participants showed a tDCS pattern that is consistent with the traditional anodal-increase and cathodal-decrease prediction, with only one participant going the other way. Therefore, perhaps the traditional anodal-increase and cathodal-decrease assumption is valid, but it applies only to a subset of participants (one-third in our case), whereas other participants (two-thirds in our case) are less sensitive to the changes in polarity. But why does the anodal-increase and cathodal-decrease rule of thumb work on these people but not others? So far there is no measurement that can tell them apart. However, it is important to note that the anodal-increase and cathodal-decrease idea was first proposed by studies done on the motor cortex because it is easy to measure and relatively easy to set a resting baseline in participants. We think the latter may be the key to explaining the diverse individual differences when tDCS is combined with a complex cognitive task; namely that a neuronal resting baseline for regions other than the motor cortex is hard to do and to monitor. As such, it is easier to tell participants to sit still and relax their muscles (and get cleaner data), it is harder to do the same with other cortical regions. Therefore, an objective way to get all participants’ task set and concentration standardized may be a useful approach to explaining different sub-categorical population differences, and possibly resolve much of the controversies and inconsistent findings in the literature. Future tDCS studies should examine each individual’s data more closely, and this issue of different subgroups reacting differently to tDCS polarity, as well the mechanisms behind such differences, require further research.

**Figure 3:**
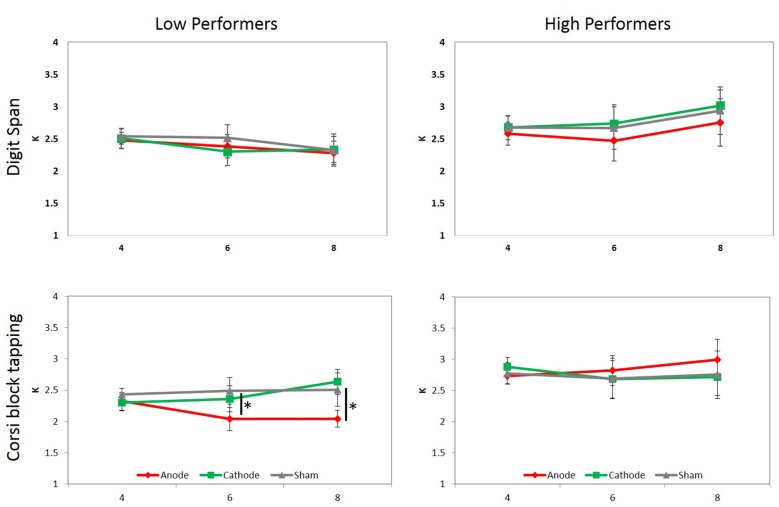
**Mean *K* values under manipulations of tDCS and set size.** Participants were divided into low and high performers by either digit span (top panel) or CBT (bottom panel) scores, and two separate three-way ANOVAs were conducted for each. A significant interaction between tDCS, set size and group was only observed when participants were divided based on their CBT performance, which was driven by lower *K* values in the anodal tDCS condition than sham and cathodal conditions under set size 6 and 8 in low performers. Asterisks denote *p* < 0.05, and error bars denote standard error of the mean.

In sum, in this study we found that visuospatial WM performance is impaired by anodal tDCS in low performers but not high performers. This pattern only holds true in the set size 6 and 8 condition, and only when participants were categorized into low and high performing group based on their CBT performance, while division based on digit span scores failed to show any systematic effects. Together, these results highlight the influence of adopting different independent baseline measures, as well as task difficulty, have on the expression of the effects of tDCS. Based on these results, future studies should: (1) choose an independent baseline measure that is within the same cognitive domain and tapping into the same neural mechanisms as the experimental dependent measure; and (2) use a medium-to-difficult level of task difficulty that is sensitive enough for any effect of tDCS to show through.

## Author Contributions

T-YH designed and performed experiment, analyzed data and wrote the article; C-HJ designed experiment and wrote the article; and PT designed experiment, analyzed data and wrote the article.

## Funding

This work was supported by grants from the Minister of Science and Technology in Taiwan to T-YH (104-2410-H-038-012-MY2), to C-HJ (101-2628-H-008-001-MY4; 103-2410-H-008-023-MY3), and to PT (104-2410-H-038-013-MY3; 104-2420-H-038-001-MY3); from Taipei Medical University to T-YH (TMU104-AE1-B08; SKH-TMU-105-05) and PT (TMU104-AE1-B07; 105TMU-SHH-20). Correspondence should be sent to T-YH (tzuyu.hsu@tmu.edu.tw) or PT (philip@tmu.edu.tw).

## Conflict of Interest Statement

The authors declare that the research was conducted in the absence of any commercial or financial relationships that could be construed as a potential conflict of interest.
